# Real‐World Impact of Steerable Sheaths for Paroxysmal Atrial Fibrillation Catheter Ablation: The INSIGHT Study

**DOI:** 10.1111/jce.16658

**Published:** 2025-04-01

**Authors:** Yasuo Okumura, Tsunesuke Kono, Akira Mizukami, Osamu Inaba, Yuji Wakamatsu, Daisuke Yamagishi, Satoshi Nakamura, Takumi Arashiro, Akira Sato, Yi Wang, Atsushi Kobori

**Affiliations:** ^1^ Nihon University Itabashi Hospital Tokyo Japan; ^2^ Nagano Chuo Hospital Nagano Japan; ^3^ Kameda Medical Center Kamogawa Japan; ^4^ Saitama Red Cross Hospital Saitama Japan; ^5^ Kobe City Medical Center General Hospital Kobe Japan; ^6^ Biosense Webster, Inc., Part of Johnson & Johnson MedTech Irvine California USA

**Keywords:** AF, atrial fibrillation, catheter ablation, real‐world study, steerable sheath

## Abstract

**Introduction:**

In atrial fibrillation (AF) ablation procedures, the CARTO VIZIGO Bidirectional Guiding Sheath has previously shown promising results in reducing fluoroscopy times without compromising clinical effectiveness or safety compared with non‐steerable sheaths.

**Methods:**

This non‐randomized, multicenter, retrospective cohort study (INSIGHT) aimed to determine the real‐world impact of the VIZIGO sheath on procedural efficiency, clinical effectiveness, and safety in paroxysmal AF (PAF) catheter ablation. Consecutive adults who underwent de novo radiofrequency (RF) ablation for PAF with a non‐VIZIGO sheath (January 2019–July 2021) or VIZIGO sheath (January 2020–July 2021) were included. Procedural characteristics, primary adverse events (PAEs), and 12‐month effectiveness (freedom from repeat ablation or recurrent atrial arrhythmia) were evaluated.

**Results:**

Of 199 patients included (mean age, 69.7 years; 59.3% male), 97 had ablation with a VIZIGO sheath and 102 with a non‐VIZIGO sheath. Significantly shorter mean times were achieved in the VIZIGO vs. non‐VIZIGO group for time to left PVI (18.1 ± 7.5 vs. 19.9 ± 5.6 min, *p* = 0.046), right PVI (16.5 ± 6.1 vs. 23.1 ± 9.9 min, *p* < 0.001), total PVI (34.6 ± 9.7 vs. 42.9 ± 11.4 min, *p* = 0.002), and fluoroscopy time (7.3 ± 10.4 vs. 18.3 ± 13.3 min, *p* < 0.001). Mean fluoroscopy dose was significantly lower (45.9 ± 112.0 vs. 139.5 ± 251.5 mGy, *p* < 0.001) with VIZIGO vs. non‐VIZIGO sheaths. Catheter stability was comparable between groups. PAE rates were similar in the VIZIGO (3.1%) and non‐VIZIGO (4.9%) groups. Freedom from repeat ablation and recurrent atrial arrhythmia at 12 months were also similar in the two groups.

**Conclusion:**

These real‐world data demonstrate that use of the VIZIGO sheath in PAF ablation procedures allows for significantly lower fluoroscopy time and dose with significantly shorter PVI isolation time, without compromising acute and long‐term effectiveness or safety.

## Graphical abstract

Reductions in fluoroscopy time and dose and shorter PVI isolation times with the VIZIGO vs. non‐VIZIGO sheath: results of the real‐world, multicenter, INSIGHT cohort study.

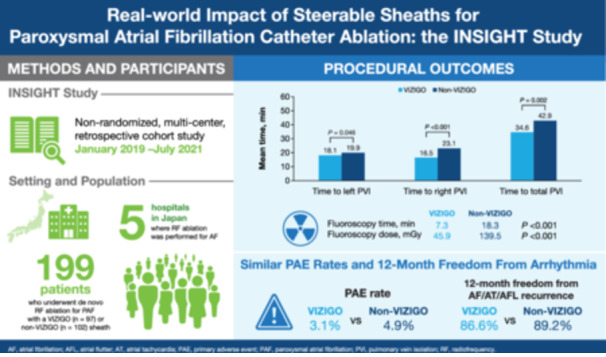

## 1 Introduction

In atrial fibrillation (AF) catheter ablation procedures, steerable sheaths provide several advantages over non‐steerable sheaths, such as improved catheter access, stability, and tissue contact at ablation sites [1, 2, 3, 4]. The use of steerable sheaths has significantly improved single‐procedure AF ablation success rates in clinical trials and case–control analyses [1, 2, 5], with recent meta‐analyses of study data concluding that steerable sheaths are associated with better efficacy and a similar safety profile compared with non‐steerable sheaths [6, 7].

X‐ray fluoroscopy is widely used to guide catheter placement in ablation procedures, but radiation exposure can pose serious health risks to patients, operators, and laboratory staff. In addition, extended time wearing lead aprons by operators and staff may lead to the development of orthopedic problems [8, 9]. Visualizable steerable sheaths that incorporate three‐dimensional (3D) electroanatomical mapping systems can improve procedural efficiency by facilitating navigation and placement of the ablation catheter and can also reduce radiation exposure [10].

The CARTO VIZIGO Bidirectional Guiding Sheath (Biosense Webster Inc., Irvine, CA) is a steerable sheath that can be visualized using the compatible CARTO 3 EP Navigation System (Biosense Webster Inc.) without fluoroscopy [11]. Several studies have demonstrated significantly reduced fluoroscopy time with the VIZIGO sheath compared with non‐steerable and non‐visualizable steerable sheaths, along with comparable or improved procedural success, clinical effectiveness, and safety; however, these studies have been limited to single‐center settings [10, 12, 13, 14, 15, 16, 17].

The objective of the INSIGHT study was to further explore the real‐world impact of the VIZIGO steerable sheath on procedural efficiency, clinical effectiveness, and safety in paroxysmal AF catheter ablation in a cohort of patients treated particularly across multiple clinics in Japan.

## 2 Methods

### 2.1 Study Design

This non‐randomized, multicenter, retrospective cohort study included consecutive adults aged ≥ 18 years who underwent a de novo radiofrequency ablation for paroxysmal AF with a radiofrequency catheter at study sites in Japan (Nihon University School of Medicine, Nagano Chuo Hospital, Kobe City Medical Center General Hospital, Kameda Medical Center, and Saitama Red Cross Hospital). Patients were included if they had undergone radiofrequency ablation with a VIZIGO sheath between January 2020 and July 2021 or without a VIZIGO sheath between January 2019 and July 2021. Patients were required to have follow‐up data through 12 months after the index ablation procedure, with the exception of patients who died before their scheduled 12‐month follow‐up visit. Key exclusion criteria included a history of persistent AF or long‐standing persistent AF at the time of the ablation procedure, any prior left atrial ablation or cardiac‐related surgery before the ablation procedure, and enrollment in a potentially confounding drug or device trial at the time of the ablation procedure or within the 12‐month follow‐up period after the procedure.

The study was conducted according to the Declaration of Helsinki, Ethical Guidelines for Life Science and Medical Research Involving Human Subjects, and the Act on the Protection of Personal Information. All included patients provided informed consent according to the Ethical Guidelines for Life Science and Medical Research Involving Human Subjects.

### 2.2 Ablation Procedures

Catheter ablation procedures were typically conducted using a THERMOCOOL SMARTTOUCH SF Catheter (Biosense Webster Inc.) in conjunction with the CARTO mapping system. Ablation procedures were conducted with either the VIZIGO sheath with or without fluoroscopy or a non‐VIZIGO sheath with fluoroscopy. PENTARAY and/or LASSO (Biosense Webster Inc.) catheters were used for mapping and verification of pulmonary vein isolation (PVI). Pulmonary veins (PVs) were isolated using a point‐by‐point ablation technique, with isolation of the PVs documented. Ablations beyond PVI, such as cavotricuspid or mitral isthmus ablations or ablations targeting non‐PV foci, were performed when these arrhythmias were either induced by burst atrial pacing or observed clinically.

### 2.3 Study Outcomes

Primary procedural efficiency outcomes for the cohort comparative analysis included fluoroscopy time and dose. Fluoroscopy details were compared further in sequential case order for the study centers. Other procedural efficiency outcomes included volume of fluid delivered, mapping time, time to PVI, total procedure time, radiofrequency time (PV and total), number of radiofrequency applications, maximum power, and catheter stability. The number of radiofrequency applications and PV radiofrequency time were also assessed by study center level. Case data from CARTO was extracted from the CARTONET cloud‐based storage and artificial intelligence analytics platform to evaluate catheter stability. The stability values used for analysis denote the mean catheter displacement during the first 90% of the ablation time for each lesion. This allowed for removal of the purposeful motion to reposition the catheter for the next lesion site.

Acute PVI outcomes included first pass PVI, return of conduction, and PVI by the end of the procedure. Longer‐term clinical effectiveness outcomes through 12 months of follow‐up included reablation; cardioversion; AF, atrial tachycardia (AT), and atrial flutter (AFL) recurrence after a 90‐day blanking period through 12 months; single‐procedure freedom from AF/AT/AFL recurrence after a 90‐day blanking through 12 months; antiarrhythmic drug status; and oral anticoagulant use. Safety outcomes included device‐ and procedure‐related primary adverse events (PAEs) within 7 days of index procedure, which included phrenic nerve paralysis/injury, stroke/cerebrovascular accident, transient ischemic attack, thromboembolism, myocardial infarction, pericarditis, vagal nerve injury, and major access complication/bleeding. Device‐ or procedure‐related death, atrioesophageal fistula, and severe pulmonary vein stenosis up to 90 days and cardiac tamponade/pericardial effusion up to 30 days post‐index procedure were also considered PAEs.

### 2.4 Statistical Analysis

Continuous data were summarized as mean ± standard deviation and tested based on Wilcoxon rank sum test for VIZIGO compared with non‐VIZIGO procedures. Categorical data were summarized as frequencies and percentages and tested based on Fisher's exact test for VIZIGO compared with non‐VIZIGO procedures. Empirical prediction plots and linear mixed‐effects models were used to estimate average fluoroscopy time and dose by study site for each ablation case sequence. Kaplan–Meier survival analysis was performed to obtain estimates and 95% confidence intervals (CI) for freedom from AF/AT/AFL recurrence after a 90‐day blanking period and reablation at any time. Statistical analyses were performed using SAS, version 3.8 (SAS Institute Inc., Cary, NC, USA).

## 3 Results

### 3.1 Baseline Patient Characteristics and Index Ablation Procedure Details

A total of 199 patients met the inclusion and exclusion criteria, with 97 patients undergoing ablation with a VIZIGO sheath and 102 with a non‐VIZIGO sheath. Of the 25 operators who performed these ablations, 15 performed procedures using both the VIZIGO and non‐VIZIGO sheaths, 3 performed procedures using only the VIZIGO sheath, and 7 performed procedures using only non‐VIZIGO sheaths. Overall, the mean age was 69.7 years and 59.3% (118/199) of the participants were male (Table 1). Baseline patient characteristics were similar between the VIZIGO and non‐VIZIGO groups.

**Table 1:** Baseline patient characteristics and index ablation procedure details.

Characteristics	All (*N* = 199)	VIZIGO (*n* = 97)	Non‐VIZIGO (*n* = 102)	*p* Value
Age, years, mean ± SD	69.7 ± 10.1	69.3 ± 11.1	70.1 ± 9.0	0.936
Male sex, *n* (%)	118 (59.3)	59 (60.8)	59 (57.8)	0.773
Weight, kg, mean ± SD	63.6 ± 12.3	63.6 ± 10.4	63.5 ± 13.8	0.727
Height, cm, mean ± SD	161.9 ± 10.3	162.5 ± 9.4	161.4 ± 11.0	0.464
BMI, kg/m^2^, mean ± SD	24.1 ± 3.5	24.1 ± 3.4	24.2 ± 3.7	0.465
Left ventricular ejection fraction, %, mean ± SD	63.5 ± 11.4	64.08 ± 9.6	63.03 ± 12.9	0.830
Left atrial diameter, mm, mean ± SD	39.16 ± 6.5	38.42 ± 6.7	39.87 ± 6.2	0.075
CHA_2_DS_2_‐VASc score, mean ± SD	2.9 ± 1.8	2.7 ± 1.8	3.1 ± 1.8	0.188
Comorbidity, *n* (%)				
Hypertension	123 (61.8)	54 (55.7)	69 (67.6)	0.108
Diabetes	32 (16.1)	12 (12.4)	20 (19.6)	0.181
Congestive heart failure	33 (16.6)	16 (16.5)	17 (16.7)	> 0.999
No vascular disease	161 (80.9)	84 (86.6)	77 (75.5)	0.050
Prior stroke	21 (10.6)	12 (12.4)	9 (8.8)	0.492
Prior transient ischemic attack	3 (1.5)	1 (1.0)	2 (2.0)	> 0.999
Prior thromboembolism	7 (3.5)	4 (4.1)	3 (2.9)	0.716
CARTO mapping system, *n* (%)	199 (100)	97 (100)	102 (100)	—
Diagnostic catheter, *n* (%)				
LASSO	20 (10.1)	8 (8.2)	12 (11.8)	0.483
DECANAV	22 (11.1)	22 (22.7)	0 (0)	< 0.001
WEBSTER CS	40 (20.1)	20 (20.6)	20 (19.6)	0.862
Soundstar	178 (89.4)	87 (89.7)	91 (89.2)	> 0.999
Pentaray	178 (89.4)	88 (90.7)	90 (88.2)	0.648
Ablation catheter, *n* (%)				
SMARTTOUCH SF	198 (99.5)	96 (99.0)	102 (100)	0.487
Unknown	1 (0.5)	1 (1.0)	0 (0)	0.487
Sheath, *n* (%)				
VIZIGO	97 (48.7)	97 (100.0)	0 (0)	< 0.001
Agilis	80 (40.2)	0 (0)	80 (78.4)	< 0.001
Other	21 (10.6)	0 (0)	21 (20.6)	< 0.001
Unknown	2 (1.0)	0 (0)	2 (2.0)	0.498
Substrate modification, *n* (%)				
Mitral isthmus	4 (2.0)	1 (1.0)	3 (2.9)	0.622
Posterior wall	30 (15.1)	15 (15.5)	15 (14.7)	> 0.999
Other	103 (51.8)	47 (48.5)	56 (54.9)	0.396

Abbreviations: BMI, body mass index; CHA_2_DS_2_‐VASc, congestive heart failure, hypertension, age ≥ 75 years (doubled), diabetes mellitus, stroke/transient ischemic attack/thromboembolism (doubled), vascular disease, age 65–74 years, sex category; SD, standard deviation.

In the non‐VIZIGO group, the Agilis NxT steerable sheath (Abbott Inc, St. Paul, MN) was used in 78.4% (80/102) of patients. The majority (68.8% [137/199]) of patients underwent ablation with substrate modification, including posterior wall in 15.1% (30/199), with no significant differences between VIZIGO and non‐VIZIGO groups (Table 1).

### 3.2 Procedural Efficiency Outcomes

**Figure 1:**
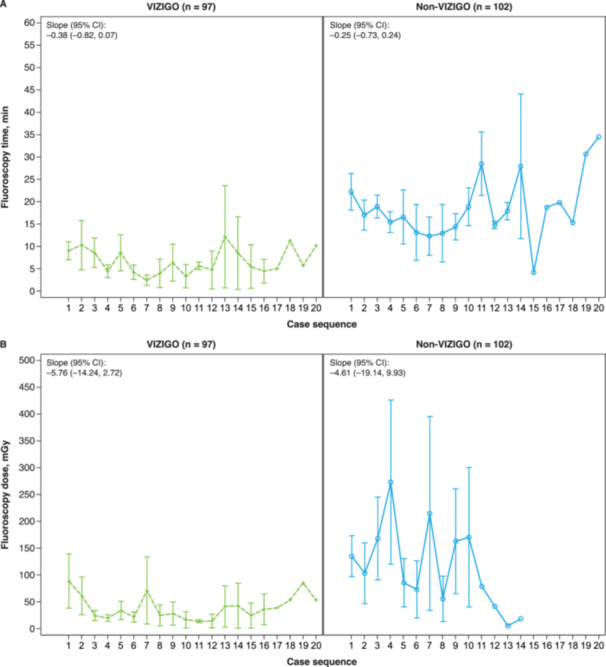
Fluoroscopy time (A) and fluoroscopy dose (B) for patients in the VIZIGO and non‐VIZIGO groups by case sequence. Data are mean ± SD averaged for each site. SD, standard deviation.

**Figure 2:**
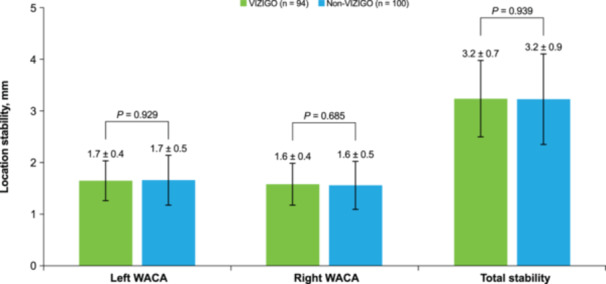
Catheter location stability for patients in the VIZIGO and non‐VIZIGO groups. Data are mean ± SD. SD, standard deviation; WACA, wide area circumferential ablation.

Procedural efficiency outcomes are summarized in Table 2. Significantly shorter mean times were achieved in the VIZIGO group vs. the non‐VIZIGO group for time to left PVI (18.1 ± 7.5 vs. 19.9 ± 5.6 min, *p* = 0.046), right PVI (16.5 ± 6.1 vs. 23.1 ± 9.9 min, *p* < 0.001), and total PVI (34.6 ± 9.7 vs. 42.9 ± 11.4 min, *p* = 0.002). The non‐VIZIGO group was associated with an average of 9.5 min longer time to PVI, adjusting for the number of radiofrequency applications (*p* < 0.001). The mean fluoroscopy time was significantly shorter (7.3 ± 10.4 vs. 18.3 ± 13.3 min, *p* < 0.001), and the mean fluoroscopy dose was significantly lower (45.9 ± 112.0 vs. 139.5 ± 251.5 mGy, *p *< 0.001) with the VIZIGO sheath compared with non‐VIZIGO sheaths (Table 2). When comparing the cases sequentially among study centers, average fluoroscopy time (slope: –0.38 [95% CI: –0.82, 0.07] vs. slope: –0.25 [95% CI: –0.73, 0.24]) and dose (slope: –5.76 [95% CI: –14.24, 2.72] vs. slope: –4.61 [95% CI: –19.14, 9.93]) decreased quicker and were consistently lower using the VIZIGO sheath as more cases were done (Figure 1). The mean PV radiofrequency time was comparable in the VIZIGO group (22.6 ± 8.3 min) and the non‐VIZIGO group (24.3 ± 8.0 min). While the mean number of radiofrequency applications was significantly higher overall in the VIZIGO group compared with the non‐VIZIGO group (129.2 ± 90.3 vs. 104.4 ± 41.7, *p* = 0.041), there were no significant differences between groups when comparing left‐ and right‐sided wide area circumferential ablation. Radiofrequency mean maximum power was significantly higher in the VIZIGO group vs. the non‐VIZIGO group for anterior (46.0 ± 3.7 vs. 41.2 ± 7.1 W, *p* < 0.001) and posterior (44.8 ± 4.6 vs. 39.6 ± 6.8 W, *p* < 0.001) applications. There were no significant differences between the VIZIGO and non‐VIZIGO groups in total catheter stability or for left‐ and right‐sided wide area circumferential ablation (Figure 2). All other procedural efficiency and PVI measures were similar in the VIZIGO and non‐VIZIGO groups.

**Table 2:** Procedural efficiency.

Parameters	All (*N* = 199)	VIZIGO (*n* = 97)	Non‐VIZIGO (*n* = 102)	*p* Value
**Procedural efficiency outcomes**,^a^ **mean ± SD**				
Fluid via catheter, mL	772.6 ± 417.6	799.2 ± 337.8	748.1 ± 481.7	0.212
Fluid via IV, mL	1007.5 ± 320.8	961.3 ± 356.4	1051.1 ± 286.5	0.631
Mapping time, min	8.5 ± 4.2	8.0 ± 4.4	9.0 ± 4.0	0.179
Time to left PVI, min	19.0 ± 6.7	18.1 ± 7.5	19.9 ± 5.6	0.046
Time to right PVI, min	19.7 ± 8.7	16.5 ± 6.1	23.1 ± 9.9	< 0.001
Time to total PVI, min	38.6 ± 11.3	34.6 ± 9.7	42.9 ± 11.4	0.002
PV radiofrequency time, min	23.5 ± 8.2	22.6 ± 8.3	24.3 ± 8.0	0.109
Radiofrequency time, min	34.9 ± 21.5	34.8 ± 26.3	35.0 ± 16.1	0.337
Procedure time, min	150.1 ± 57.3	149.4 ± 61.1	150.7 ± 53.9	0.637
Fluoroscopy time, min	12.8 ± 13.2	7.3 ± 10.4	18.3 ± 13.3	< 0.001
Fluoroscopy dose, mGy	90.4 ± 196.6	45.9 ± 112.0	139.5 ± 251.5	< 0.001
Number of radiofrequency applications	116.2 ± 70.3	129.2 ± 90.3	104.4 ± 41.7	0.041
Left WACA	46.1 ± 17.9	47.4 ± 18.8	44.9 ± 17.0	0.185
Right WACA	45.9 ± 15.5	45.2 ± 16.6	46.7 ± 14.5	0.398
Maximum power, W				
Anterior	43.5 ± 6.2	46.0 ± 3.7	41.2 ± 7.1	< 0.001
Posterior	42.1 ± 6.4	44.8 ± 4.6	39.6 ± 6.8	< 0.001
**Acute PVI outcomes, *n* (%)**				
All PVs isolated by end of procedure	199 (100)	97 (100)	102 (100)	‐‐‐

Abbreviations: PV, pulmonary vein; PVI, pulmonary vein isolation; SD, standard deviation; WACA, wide area circumferential ablation.

^a^ With available data.

**Figure 3:**
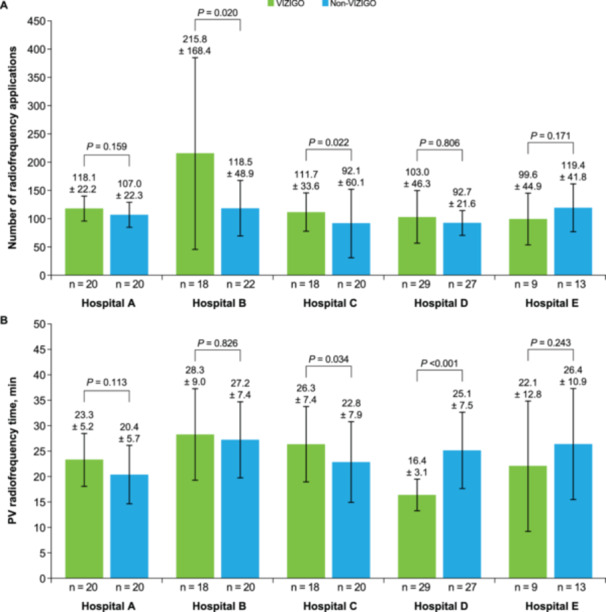
Number of radiofrequency applications (A) and PV radiofrequency time (B) by study site. Data are mean ± SD. PV, pulmonary vein; SD, standard deviation.

The mean number of radiofrequency applications and PV radiofrequency time varied by study site (Figure 3). At two study sites, the number of radiofrequency applications was significantly higher in the VIZIGO than in the non‐VIZIGO group (*p *≤ 0.022; Figure 3A). At the remaining three study sites, there was no significant difference in the number of radiofrequency applications between the VIZIGO and non‐VIZIGO groups. PV radiofrequency time was significantly longer in the VIZIGO than in the non‐VIZIGO group at one study site (*p* = 0.034), was significantly shorter in the VIZIGO than in the non‐VIZIGO group at another site (*p* < 0.001), and did not differ significantly between the VIZIGO and non‐VIZIGO groups at the remaining three sites (Figure 3B).

For the 15 operators who performed procedures using both the VIZIGO and non‐VIZIGO sheaths, mean fluoroscopy time (13.6 ± 13.9 min), radiofrequency time (31.5 ± 12.3 min), and time to total PVI (34.6 ± 8.6) were similar to those observed for the overall group of operators (12.8 ± 13.2, 34.9 ± 21.5, and 38.6 ± 11.3 min, respectively).

### 3.3 Safety

Device/procedure‐related PAE rates were similar in the VIZIGO and non‐VIZIGO groups (3.1% [3/97] vs. 4.9% [5/102], *p* = 0.722; Table 3). In the VIZIGO group, PAEs were reported in 3 patients and comprised cardiac tamponade/pericardial effusion (1 patient), major vascular access/bleeding (2 patients), and thromboembolism (1 patient, who also experienced major vascular access/bleeding), while in the non‐VIZIGO group, PAEs were reported in 5 patients and comprised cardiac tamponade/pericardial effusion (1 patient) and major vascular access/bleeding (4 patients). There were no reports of atrioesophageal fistula, phrenic nerve paralysis/injury, severe pulmonary vein stenosis, stroke, transient ischemic attack, myocardial infarction, pericarditis, or vagal nerve injury. No procedure‐related deaths occurred.

**Table 3:** Clinical outcomes.

Parameters, *n* (%)	All (*N* = 199)	VIZIGO (*n* = 97)	Non‐VIZIGO (*n* = 102)	*p* Value
**Safety**				
Any PAE	8 (4.0)	3 (3.1)	5 (4.9)	0.722
Cardiac tamponade/pericardial effusion	2 (1.0)	1 (1.0)	1 (1.0)	> 0.999
Major vascular access/bleeding	6 (3.0)	2 (2.1)	4 (3.9)	0.683
Thromboembolism	1 (0.5)	1 (1.0)	0 (0)	0.490
Atrioesophageal fistula	0 (0)	0 (0)	0 (0)	‐‐‐
Phrenic nerve paralysis/injury	0 (0)	0 (0)	0 (0)	‐‐‐
Severe pulmonary vein stenosis	0 (0)	0 (0)	0 (0)	‐‐‐
Device/procedure‐related death	0 (0)	0 (0)	0 (0)	‐‐‐
Stroke/cerebrovascular accident	0 (0)	0 (0)	0 (0)	‐‐‐
Myocardial infarction	0 (0)	0 (0)	0 (0)	‐‐‐
Transient ischemic attack	0 (0)	0 (0)	0 (0)	‐‐‐
Pericarditis	0 (0)	0 (0)	0 (0)	‐‐‐
Vagal nerve injury	0 (0)	0 (0)	0 (0)	‐‐‐
Unknown	0 (0)	0 (0)	0 (0)	‐‐‐
**Effectiveness at 12‐month follow‐up**				
Repeat ablation				0.408
Yes	17 (8.5)	10 (10.3)	7 (6.9)	
No	175 (87.9)	85 (87.6)	90 (88.2)	
Unknown	7 (3.5)	2 (2.1)	5 (4.9)	
Cardioversion				0.519
Yes	6 (3.0)	4 (4.1)	2 (2.0)	
No	180 (90.5)	88 (90.7)	92 (90.2)	
Unknown	13 (6.5)	5 (5.2)	8 (7.8)	
AF/AT/AFL recurrence				0.410
Yes	27 (13.6)	13 (13.4)	14 (13.7)	
No	159 (79.9)	80 (82.5)	79 (77.5)	
Unknown	13 (6.5)	4 (4.1)	9 (8.8)	
AAD status				
Not on AAD	126 (63.3)	60 (61.9)	66 (64.7)	0.769
Placed on previous AAD at same dose	29 (14.6)	14 (14.4)	15 (14.7)	> 0.999
Placed on previous AAD at higher dose	5 (2.5)	1 (1.0)	4 (3.9)	0.369
Placed on new AAD	27 (13.6)	16 (16.5)	11 (10.8)	0.301
OAC use				0.708
Yes	135 (67.8)	63 (64.9)	72 (70.6)	
No	50 (25.1)	27 (27.8)	23 (22.5)	
Unknown	14 (7.0)	7 (7.2)	7 (6.9)	

Abbreviations: AAD, antiarrhythmic drug; AF, atrial fibrillation; AFL, atrial flutter; AT, atrial tachycardia; OAC, oral anticoagulant; PAE, primary adverse event.

### 3.4 Clinical Effectiveness

**Figure 4:**
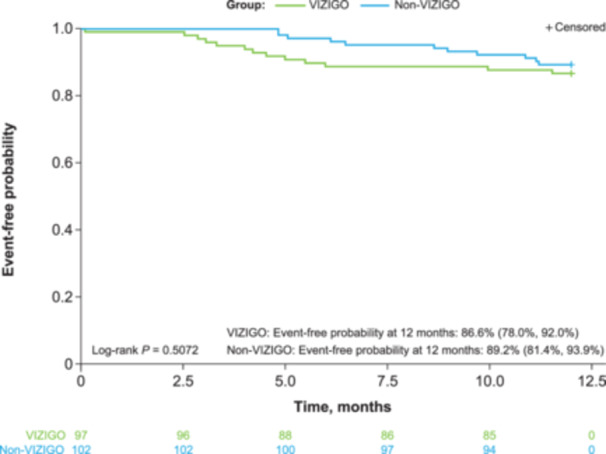
Kaplan–Meier single procedure survival from AF/AT/AFL recurrence.* *Single‐procedure survival was defined as freedom from recurrence after a 90‐day blanking period and reablation at any time. AF, atrial fibrillation; AFL, atrial flutter; AT, atrial tachycardia.

Effectiveness outcomes are summarized in Table 3. After 12 months of follow‐up, clinical effectiveness outcomes, including repeat ablation, cardioversion, and AF/AT/AFL recurrence, were similar in the VIZIGO and non‐VIZIGO groups. Kaplan–Meier estimates of single‐procedure freedom from AF/AT/AFL recurrence at 12 months were 86.6% (95% CI: 78.0%, 92.0%) in the VIZIGO group and 89.2% (95% CI: 81.4%, 93.9%) in the non‐VIZIGO group (*p* = 0.5072, Figure 4). The 12‐month success rate (85.1%) was similar for the group of operators (*n* = 15) who used both VIZIGO and non‐VIZIGO sheaths. Most patients in the VIZIGO and non‐VIZIGO groups were not on an antiarrhythmic medication at 12 months (61.9% [60/97] and 64.7% [66/102], respectively), while most were on oral anticoagulant therapy at 12 months (64.9% [63/97] and 70.6% [72/102], respectively).

## 4 Discussion

In the current study, use of the VIZIGO sheath in paroxysmal AF ablation procedures significantly shortened fluoroscopy time and significantly lowered fluoroscopy dose compared with use of a non‐VIZIGO sheath. We observed no differences in procedural safety or effectiveness, with a low incidence of device/procedure‐related PAEs in both the VIZIGO and non‐VIZIGO groups (3.1% and 4.9%, respectively) and a > 85% single‐procedure freedom from AF/AT/ATL recurrence through 12 months in both the groups.

The VIZIGO design allows for sheath visualization on the CARTO map during a procedure without depending solely on fluoroscopy. Accordingly, the results of the current study are consistent with previous studies that have reported a significantly lower fluoroscopy dose and/or time with the VIZIGO sheath vs. fixed or other steerable sheaths [10, 12, 13, 14, 15, 16, 17]. While the rate of procedures that did not employ fluoroscopy was not recorded in the current cohort, previous studies have confirmed that VIZIGO can be incorporated into zero fluoroscopy workflows. Bertini et al. first described zero fluoroscopy workflow in two cases of AF ablation using the VIZIGO sheath under 3D electroanatomic mapping and intracardiac echocardiography guidance [18]. Subsequently, in a US single‐center study, 99% of 142 procedures that used the VIZIGO sheath were performed without fluoroscopy [13], while at a Hungarian clinic, 88% of 50 VIZIGO procedures used no fluoroscopy after the transseptal puncture [14]. In contrast, non‐visualized steerable sheaths, such as the Agilis sheath used in nearly 80% of procedures in the non‐VIZIGO group in the current study, cannot be incorporated into zero fluoroscopy workflows. Overall, results from this and previous studies support use of the VIZIGO sheath in minimal fluoroscopy workflows and in the transition to zero fluoroscopy procedures if desired [13].

Another advantageous feature of the VIZIGO sheath is that it is bidirectional, with deflections of 180° in both directions, which allows for greater access to sites beyond the PV [11].

In the current study, PVI was achieved by the end of the procedure in all the patients regardless of sheath type, although time to PVI was significantly shorter in the VIZIGO group, at 35 min, vs. 43 min in the non‐VIZIGO group. The overall procedure time was not reduced with the VIZIGO sheath in this study, possibly due to the time needed to generate a 3D matrix of the right atrium for visualization of the sheath before proceeding to the left atrium. It is also possible that operators may have had a learning curve as they adjusted to the new VIZIGO technology.

An analysis of the number of radiofrequency applications and PV radiofrequency time by study site demonstrated some differences across the hospitals included in this study. The number of radiofrequency applications varied substantially but was higher with the VIZIGO sheath than with non‐VIZIGO sheaths at four of the five participating hospitals, although this difference did not reach statistical significance at two of those hospitals. This finding suggests that ablation of additional targets was pursued more frequently with the VIZIGO than with non‐VIZIGO sheaths. The differences in the number of radiofrequency applications and PV radiofrequency time may reflect differences in operator experience or hospital protocols; nevertheless, the trends observed with the VIZIGO compared with the non‐VIZIGO sheath were generally consistent across study sites in this multicenter study.

Our findings showed that using the VIZIGO sheath with reduced fluoroscopy and alternative imaging enabled operators to achieve similar catheter stability as with non‐VIZIGO sheaths with fluoroscopy. Other studies have reported greater catheter stability with the VIZIGO sheath vs. non‐steerable sheaths [13, 15]. While the small studies conducted so far have not shown a difference in long‐term clinical outcomes with VIZIGO vs. other sheaths, catheter and contact‐force stability is known to be a predictor of 12‐month postablation success [19].

The limitations of this study include the non‐randomized retrospective study design, lack of adjustment for confounding factors, and limited generalizability to patients outside of Japan. This study was not aimed to compare the costs between using VIZIGO vs. non‐VIZIGO sheaths. As these costs can vary by region, future evaluations may be beneficial to understand cost impact.

## 5 Conclusions

These real‐world data demonstrate that when treating paroxysmal AF by radiofrequency ablation, use of the VIZIGO sheath can significantly shorten fluoroscopy time and reduce fluoroscopy dose, thereby alleviating the burden of heavy protective equipment, while improving procedural efficiency without compromising safety or acute and longer‐term clinical effectiveness of AF ablation.

## Ethics Statement

The study was conducted according to the Declaration of Helsinki, Ethical Guidelines for Life Science and Medical Research Involving Human Subjects, and the Act on the Protection of Personal Information.

## Consent

All included patients provided informed consent according to the Ethical Guidelines for Life Science and Medical Research Involving Human Subjects.

## Conflicts of Interest

Yasuo Okumura received research grants unrelated to this study from Johnson & Johnson K.K. and Biosense Webster Inc.; scholarship funds from Nippon Boehringer Ingelheim, remuneration from Daiichi‐Sankyo, AstraZeneca, Bayer Healthcare, Bristol Myers Squibb, and Johnson & Johnson K.K.; and belongs to the endowed departments of Boston Scientific Japan, Biotronik Japan, Abbott Medical Japan, Japan Lifeline, and Medtronic Japan. Akira Mizukami received speaking fees from Johnson & Johnson K.K., Medtronic Japan Co. Ltd., and Abbott Japan LLC. Osamu Inaba received speaking fees and research funding from Johnson & Johnson K.K., Boston Scientific Japan K.K., and Medtronic Japan Co. Ltd. Daisuke Yamagishi received speaking fees from Johnson & Johnson K.K., Toray Industries Inc., Toray Medical Inc., Japan Lifeline Inc., Synaptic Medical Japan Inc., and Nihon Kohden Inc.; grants from Biosense Webster Inc., part of Johnson & Johnson MedTech. Yi Wang is a contractor providing biostatistical support for Biosense Webster Inc., part of Johnson & Johnson MedTech. Atsushi Kobori receives speaking fees from Johnson & Johnson K.K., Boston Scientific Japan, Abbott Medical Japan, Japan Lifeline, and Medtronic Japan. The other authors declare no conflicts of interest.

## Data Availability

The data that support the findings of this study are available from the corresponding author upon reasonable request. Johnson & Johnson MedTech has an agreement with the Yale Open Data Access Project to serve as the independent review panel for evaluation of requests for clinical study reports and participant‐level data from investigators and physicians for scientific research that will advance medical knowledge and public health. Requests for access to the study data can be submitted through the Yale Open Data Access Project site at http://yoda.yale.edu.
